# TFPI-2 Protects Against Gram-Negative Bacterial Infection

**DOI:** 10.3389/fimmu.2018.02072

**Published:** 2018-09-11

**Authors:** Mohamad N. Ali, Gopinath Kasetty, Malin Elvén, Saud Alyafei, Sandra Jovic, Arne Egesten, Heiko Herwald, Artur Schmidtchen, Praveen Papareddy

**Affiliations:** ^1^Division of Dermatology and Venereology, Department of Clinical Sciences Lund, Lund University, Lund, Sweden; ^2^Division of Respiratory Medicine and Allergology, Department of Clinical Sciences Lund, Lund University, Lund, Sweden; ^3^Division of Infection Medicine, Department of Clinical Sciences Lund, Lund University, Lund, Sweden; ^4^Dermatology, LKCMedicine, Nanyang Technological University, Singapore, Singapore; ^5^Department of Biomedical Sciences, Copenhagen Wound Healing Center, Bispebjerg Hospital, University of Copenhagen, Copenhagen, Denmark

**Keywords:** TFPI-2, antimicrobial peptide, bacteria, complement, immunoglobulins, sepsis

## Abstract

Tissue factor pathway inhibitor-2 (TFPI-2) has previously been characterized as an endogenous anticoagulant. TFPI-2 is expressed in the vast majority of cells, mainly secreted into the extracellular matrix. Recently we reported that EDC34, a C-terminal peptide derived from TFPI-2, exerts a broad antimicrobial activity. In the present study, we describe a previously unknown antimicrobial mode of action for the human TFPI-2 C-terminal peptide EDC34, mediated via binding to immunoglobulins of the classes IgG, IgA, IgE, and IgM. In particular the interaction of EDC34 with the Fc part of IgG is of importance since this boosts interaction between the immunoglobulin and complement factor C1q. Moreover, we find that the binding increases the C1q engagement of the antigen-antibody interaction, leading to enhanced activation of the classical complement pathway during bacterial infection. In experimental murine models of infection and endotoxin challenge, we show that TFPI-2 is up-regulated in several organs, including the lung. Correspondingly, *TFPI-2*^−/−^ mice are more susceptible to pulmonary *Pseudomonas aeruginosa* bacterial infection. No anti-coagulant role of TFPI-2 was observed in these models *in vivo*. Furthermore, *in vivo*, the mouse TFPI-2-derived C-terminal peptide VKG24, a homolog to human EDC34 is protective against systemic *Escherichia coli* bacterial infection. Moreover, in sputum from cystic fibrosis patients TFPI-2 C-terminal fragments are generated and found associated with immunoglobulins. Together our data describe a previously unknown host defense mechanism and therapeutic importance of TFPI-2 against invading Gram-negative bacterial pathogens.

## Introduction

The exploration of immuno-modulating proteins and peptides has attracted a significant interest within the past years (1, 2). To this end, techniques such as phage display (3, 4) and peptide array technologies (5) had been developed that allow the detection of potential interactions between these peptides and their potential interaction partners. Notably, the mammalian immune system is in the focus of such analyses as there are many reports showing that it can by modulated by a variety of exogenous peptides. During million years of evolution the mammalian immune system has evolved into a complex and self-regulating network comprised by many components that orchestrate with each other to ascertain maximum protection for the host (6). However, some microorganisms have succeeded to manipulate the host response by deceiving the immune defense system and crossing the firewall of protection. Though bacteria can very easily adapt to new hostile environments, there have been only be a few examples showing that they can also counteract the activity of antimicrobial peptides (AMPs). Thus, AMPs demonstrate a significant potential as novel therapeutic agents. Many researchers have therefore extensively investigated different classes of endogenous peptides derived for instance from mammalian neutrophils, i.e., cathelicidins and defensins (7). AMPs are found in all life forms including bacteria, fungi, plants, invertebrates, and vertebrates (8). They are essential components of the innate immune system (9), as they help to ensure the maximum protection for the host against pathogens and invading microbes (10, 11). In addition to their involvement in direct extermination of various microorganisms, AMPs can mediate diverse immunomodulatory responses (12, 13). These include stimulation of macrophages, lymphocytes and neutrophils (9), induction of angiogenesis (14), triggering in wound healing (15), and neutralizing lipopolysaccharide endotoxins derived from Gram-negative bacteria (16).

During the past decade there have been several findings on HDPs generated from proteins involved in coagulation including thrombin (17–19), HCAII (20), TFPI-1 (21) among others. There are also host derived peptides generated through proteolytic cleavage by bacterial proteases, as recently discovered in the case of *Pseudomonas aeruginosa* (22). *P. aeruginosa* infect by several additional strategies, targeting matrix remodeling, impressively proven by the *TIMP-1*^−/−^ mice and their resistance to infection by this particular pathogen. Importantly, the protective role in the case of *TIMP1*^−/−^ mice was accompanied by increased inflammatory and complement-dependent immune regulation (23). Structurally related, to an extent of approximately 50% on both nucleotide and amino acid level to TFPI-1, is it's sister protein TFPI-2 also called Matrix Serine Proteinase Inhibitor (MSPI) and Placental Protein 5 (PP5) (24–26). TFPI-2 exerts not only antimicrobial properties against Gram-negative infection (27)—the protein is also present in skin and the C-terminal region have shown to be up-regulated at the edges of both acute and chronic wounds (28). Importantly, TFPI-2 has previously been shown to inhibit several MMPs, both indirectly and through protein-protein interactions. The importance of the MMP inhibitory capabilities of TFPI-2 during dysfunctional matrix turnover as in arthritis, atherosclerosis and cancer are getting increased attention (29–31). The possibility to modify local concentrations of MMPs and their inhibitors has a large therapeutic potential in many immunopathologies, including those seen during infections (32).

Previous studies have shown that complement activation can be amplified by targeting certain proteins such as complement factor C3 (33). Elvington et al. for instance described in 2012 a method showing that complement activation can be used as cancer therapy. To this end the authors employed a fusion protein of a complement receptor and an IgG Fc fragment to improve the outcome of mAb therapy in a murine metastatic cancer model (33). In another study, we found that EDC34, a peptide derived from human TFPI-2, enhances the binding of C1q to bacterial surfaces. Moreover EDC34 is able to up regulate the antimicrobial activity of C3a, which in turn can cause amplification of the classical complement system pathway (27). These findings further reveal that EDC34 has a significant capacity to eradicate in particular Gram-negative bacteria *in vivo* (27, 28). Based on these results, we therefore decided to investigate the mode of action of C-terminal TFPI-2 region under infectious disease conditions. Our finding demonstrates that EDC34 is capable of binding to immunoglobulins (IgA, IgG, IgE, and IgM), by specifically engaging the Fc region of IgG. These finding were confirmed in *in vivo* infection studies using a peptide derived from the C-terminal part of murine TFPI-2 and were further underlined by employing TFPI-2^−/−^ mice experiments. Together our results suggest an important role of the C-terminal TFPI-2 region in boosting the host defense against invading pathogens.

## Materials and methods

### Peptides and microorganisms

The peptides listed in Table 1 were synthesized by Biopeptide Co., San Diego, CA. The purity (>95%) of these peptides was confirmed by mass spectral analysis (MALDI-ToF Voyager). The bacterial isolate *Escherichia coli* ATCC 25922 was obtained from the American Type Culture Collection and Xen41 was obtained form PerkinElmer. Mouse VKG24 peptide was synthesized by Ontores (Shanghai, China).

**Table 1:** C-terminal human TFPI-2 derived peptides.

**TFPI-2 derived peptides**
EDC34:	EDCKRACAKALKKKKKMPKLRFASRIRKIRKKQF
AKA27:	— —-AKALKKKKKMPKLRFASRIRKIRKKQF
AKA27 (S1):	— —-AKALSSSSSMPKLRFASRIRKIRKKQF
AKA27 (S2):	— —-AKALKKKKKMPKLRFASSISSISSSQF
AKA27 (S3):	— —-AKALSSSSSMPKLRFASSISSISSSQF
LKK22:	LKKKKKMPKLRFASRIRKIRKK
AKA15:	AKALKKKKKMPKLRF
LRF15:	LRFASRIRKIRKKQF
DAA14:	DAAQEPTGNNAET

*The colored letters indicate a substitution of either lysine or arginine into serine residue. DAA14 is derived from the N-terminal region of human TFPI-2*.

### Radial diffusion assay (RDA)

*Escherichia coli* was grown to mid-logarithmic phase in 10 ml (3% w/v) of trypticase soy broth (TSB). Bacteria were washed and re-suspended in 10 mM Tris, pH 7.4 to obtain a dilution of 1% of bacterial suspension (1–2 × 10^9^ colony forming units cfu/ml). Subsequently, 6.6 μl of bacteria were added to 15 ml of melted underlay agarose gel consisting of 0.03% (w/v) TSB, 1% (w/v) low electroendosmosis type (EEO) agarose and 0.02% (v/v) Tween 20 (Sigma) with or without 150 mM NaCl. The mixture was poured to a 144 mm sterile petri dish and left to solidify. After that, 4 mm diameter wells were punctured and 100 μM peptides were added to each well. Plates were incubated for 3 h at 37°C and then covered by adding 15 ml of over-lay gel consisting of 6% TSB and 1% Low-EEO agarose in Millipore water. After 16–24 h of incubation at 37°C, the peptides diffusion around each well formed the zone of inhibition, which corresponded to the peptide's antimicrobial activity.

### Viable count assay (VCA)

*Escherichia coli* bacteria were grown to mid-logarithmic phase in 10 ml of (3% w/v) Todd-Hewitt (TH), bacteria were washed and diluted in buffer 10 mM Tris, pH 7.4, containing 150 mM NaCl, either alone or with the presence of human 20% citrate plasma, lepirudin, heparin, EDTA plasma, or serum. 1–2 × 10^6^ cfu/ml bacteria were incubated in a total volume of 50 μl with the indicated concentrations of C-terminal TFPI-2 derived peptides for 2 h at 37°C. Alternatively, 1–2 × 10^6^ cfu/ml of *E. coli* bacteria in the presence or absence of EDC34 were incubated either in PBS alone or PBS supplemented with 25% of human citrated plasma, immunoglobulins depleted citrated plasma, or immunoglobulins depleted citrated plasma supplemented with 10 μg of IgG/μl, for 2 h at 37°C. Serial of dilutions of the reaction mixture were plated on TH agar plates, followed by the incubation at 37°C overnight. The number of colony forming units was determined as a percentage to control sample.

### SDS PAGE and immunoblotting

Three μg of IgG or IgA or IgE or IgM were incubated in 20 μl of buffer (10 mM Tris, 150 mM NaCl, pH 7.4), either alone or with 0.5 μg (6 μM) of EDC34 for 1 h at 37°C. Samples were diluted in SDS buffer and analyzed under non-reducing conditions by SDS-PAGE on Criterion TGX Any kD Gel (BioRad). Alternatively, EDC34 (6 μM) was incubated either alone or in PBS containing 20% serum, citrate, lepirudin, EDTA plasma at different time points (15, 30, 60, and 120 min). Samples were analyzed under non-reducing conditions.

Six μM of EDC34 was either incubated alone or with IgGs in 10 mM Tris, 150 mM NaCl, pH 7.4 buffer, under similar concentrations for 15, 30, 60, 120 min or 24 h at 37°C. In another experiment, 6 μM of EDC34 was pre-incubated with 50 μM of TCEP (tris(2-carboxyethyl)phosphine) for 30 min at room temperature before IgG (6 μM) was added. The mixture was further incubated for 24 h at 37°C. In another experiment, 6 μM of IgG or Fab-IgG or Fc-IgG were incubated with or without 6 μM of EDC34 in 10 mM Tris, 150 mM NaCl, pH 7.4 buffer for 30 min at 37°C. Proteins and peptides were transferred to nitrocellulose membranes (Hybond-C), blocked by 5% (w/v) skimmed milk, washed and incubated with rabbit polyclonal antibodies to CAK27 or rabbit polyclonal antibodies to IgG (or 1:1,000) (Dako) or rabbit polyclonal antibodies to C1q (or 1:1,000) (Dako). Polyclonal swine anti-rabbit immunoglobulin secondary HRP antibody (1:1,000) was used for detection and an enhanced chemiluminescent Substrate (LumiGLO) developing system (Upstate cell signaling solutions).

### Pull down assay

In a total volume of 500 μl PBS buffer, 2 μM of EDC34, and 8 μM of IgG were incubated for 24 h at 37°C. Subsequently, *E. coli* bacteria (1–2 × 10^6^ cfu/ml) were added, and then the mixture was incubated for additional 1 h. In a parallel experiment, the bacteria in the presence of absence of EDC34 were incubated for 30 min at 37°C before IgG was added for 1 h at 37°C. Samples were then centrifuged, bacterial pellets were washed twice with PBS and then re-suspended in 360 μl of 0.1 M glycine, pH 2.0 to elute the bound proteins. The pH of the eluted material was raised to 7.5 with 1 M Tris. Thereafter, 100 μl of 100% TCA were added to precipitate the eluted proteins followed by incubation for 10 min at −20° C followed by a centrifugation step for 20 min at 15,000 × g (4°C). The pellets were washed with acetone and left to air dry before they were dissolved in loading dye and analyzed under non-reducing conditions by SDS-PAGE, followed by Western blot analysis.

### Immunoglobulin depletion

The experiments were performed according to the manufacture's instructions. Briefly, total albumins and (>98%) and immunoglobulins (IgG, IgA, IgM, IgE, and IgD >99%) were depleted in human citrated plasma using PureProteome^TM^ Human Albumin/Immunoglobulin Depletion Kit (Millipore).

### Surface plasmon resonance

IgG was immobilized (15 μg/mL) on a CM5 sensor chip via amine coupling in a 10 mM sodium acetate buffer (pH 4). A blank immobilization was performed in flow cell one. EDC34 was injected at different concentrations (25–400 nM) at a flow rate of 30 μl/min and a temperature of 25°C over the flow cells using a running buffer containing 10 mm Hepes, 150 mm NaCl, 0.005% surfactant P20, and 3.4 mM EDTA (pH 7.5). Regeneration of the sensor surface was conducted in 0.5 M sodium chloride. The analysis was performed using a Biacore X100 instrument (GE Healthcare, Uppsala, Sweden) and the data was fitted using a 1:1 binding model.

### Generation of TFPI-2 knock out mice

TFPI-2 conditional knockout mice were generated by Deltagen (San Mateo, CA, USA). Male chimeric mice were mated with C57BL/6J female mice, and F1 heterozygous mice *TFPI-2*^+/−^, were mated with each other to yield complete knockout mice *TFPI-2*^−/−^ (Data sheet 1). All experiments were performed facilitating female and male mice at the age between of 6 and 8 weeks according to protocols approved by the Animal Ethics Committee, Lund, Sweden. Animals were housed under standard conditions of light and temperature and had free access to standard laboratory chow and water.

### *E. coli* mouse infection model

*Escherichia coli* ATCC 25922 bacteria were grown to reach mid-exponential phase (OD_620_~0.4), washed twice in PBS and then diluted in PBS. Balb/c mice (7 weeks, *n* = 6 per group) were intraperitoneally (i. p) injected with 100 μl (8–8.5 × 10^8^ cfu/ml) of *E. coli*. One hour after bacterial injection, VKG24 (500 μg per mouse) or PBS buffer alone were injected (i. p). Mice were sacrificed after 8 h post-infection and cfu were evaluated in blood, lung, spleen, kidney and liver.

### Reactive oxygen species (ROS) production assay

AIN-93M-Purified diet (ENVIGO) was provided for 1 week to mice, after 8 h post-infection mice were anesthetized and L-021 dye 25 mg per kg (Wako) was injected subcutaneously (s. c.), after 10–12 min of incubation. Reactive oxygen species (ROS) production was detected by measuring fluorescence in radiance (p/sec) for control and treated mice using IVIS® Spectrum (version 4.4, Caliper Life Sciences). Imaging was performed in groups of 2 and 3 animals at a time.

### *In vivo* TFPI-2 expression

*Escherichia coli* ATCC 25922 bacteria were grown to mid-exponential phase (OD620~0.5), harvested, washed in PBS and diluted in the same buffer to 3–4 × 10^8^ cfu/ml. Two hundred microliter of the bacterial suspension was injected intraperitoneally (i. p) into C57BL/6 mice. In another experiment, C57BL/6 mice were i. p. injected with 8 mg/kg of *E. coli* O111:B4 LPS (Sigma). In both cases, for evaluation of TFPI-2 expression, mice were sacrificed 0, 1, 2, 4, 6, and 12 h post-infection, and lung, brain, spleen, liver, kidney, small, and large intestine were harvested in trizol reagent. Before isolating RNA all organs were stored at −80°C.

### *P. aeruginosa* mouse infection model

Animals were housed under standard conditions of light and temperature, with access to chow and water *ad libitum*. *Pseudomonas aeruginosa* Xen41 bacteria were grown to logarithmic phase (OD_620_~0.5), harvested, washed in PBS, pH 7.4 and diluted in PBS to either 3–4 × 10^8^ cfu/ml, and kept on ice until injection. Fifty microliter of the bacterial suspension was administered intra nasally into mice. The survival data were obtained by following the animals daily up to 7 days monitoring. Mice reaching the pre-defined endpoint criteria were sacrificed and counted as non-survivors. For bacterial dissemination, mice were sacrificed after 12 h and cfu were determined in BALF and lung.

### Cytokine assay

The cytokines IL-6, IL-10, MCP-1, IFN-γ, TNF-α were measured in plasma and BAL fluid from mice intra-nasally infected with *P. aeruginosa* using the Cytometric bead array (CBA) Mouse Inflammation Kit # 552364 (Becton Dickinson AB) according to the manufacturer's instructions on a FACSCalibur flow cytometer (Becton Dickinson AB). All plasma samples were stored at −80°C before the analysis.

### Statistical analyses

Values are shown as mean with SEM. For statistical evaluation of two experimental groups, one-way with Tukey's multiple comparisons post-test was used and for comparison of survival curves the Mantel-Cox's test. Viable count and radial diffusion assay data are presented as mean with SD. All statistical evaluations were performed using the GraphPad Prism software 7.0 with ^*^p < 0.05, ^**^p < 0.01, ^***^ < 0.001, ^****^p < 0.0001, and ns, not significant.

## Results

### Functional characterization of EDC34 under *ex vivo* conditions

**Figure 1:**
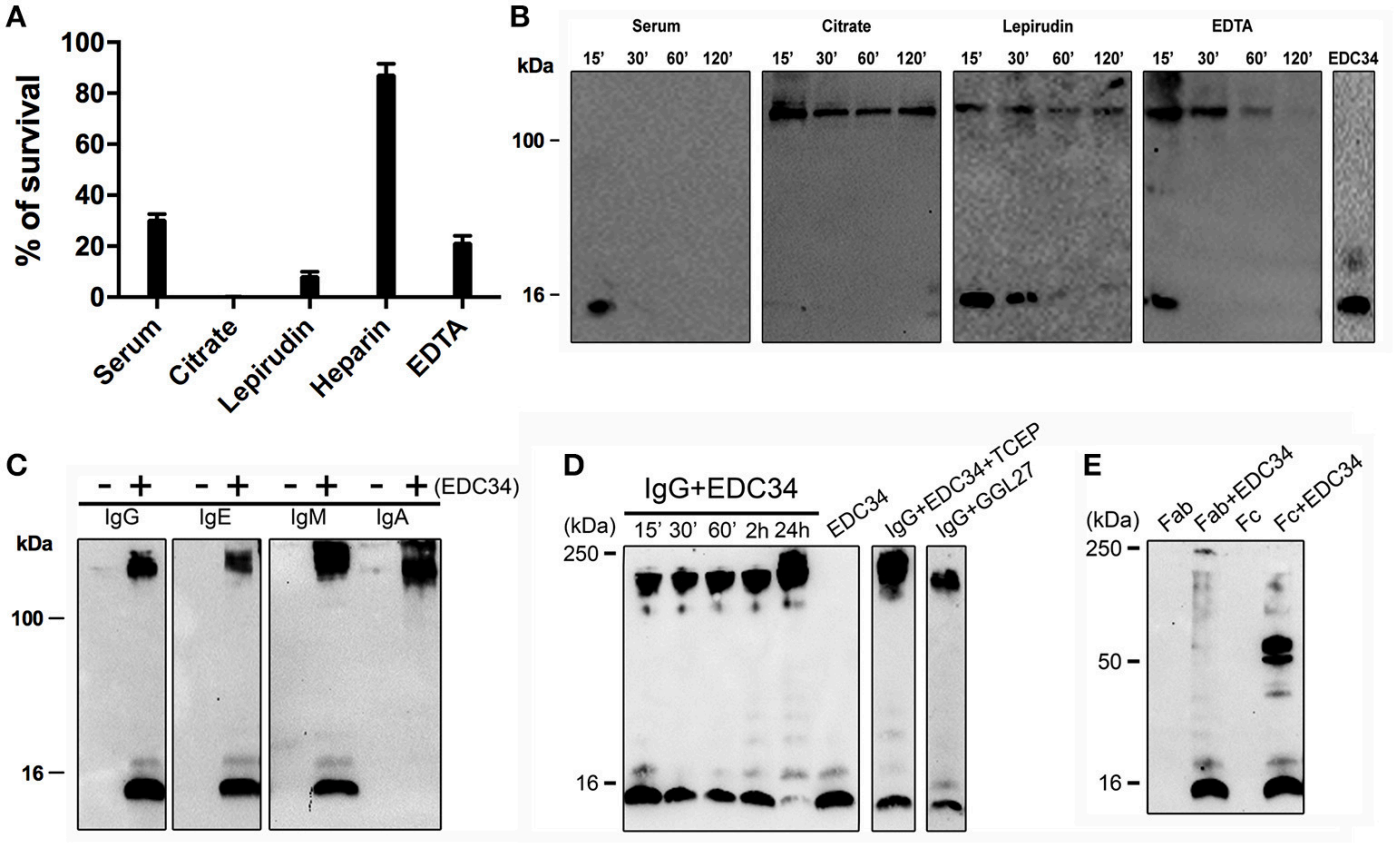
EDC34 binds to immunoglobulins. **(A)** The antimicrobial activity of EDC34 (6 μM) was tested against *E. coli* bacteria in 10 mM Tris, 150 mM NaCl, pH 7.4 buffer, containing 20% of serum or citrate or lepirudin or heparin or EDTA plasma. Mean with SD are shown (*n* = 3). **(B)** 6 μM of EDC34 were incubated in PBS alone (control) or a buffer containing 20% of serum, citrate, lepirudin, or EDTA plasma for 15 or 30 or 60 or 120 min at 37°C followed by electrophoretic analysis. EDC34 (1 μg) in 10 mM Tris was served control. **(C)** 0.5 μg of EDC34 were incubated with 3 μg of IgG, IgA, IgE, or IgM dissolved in 10 mM Tris, 150 mM NaCl, pH 7.4 buffer, for 1 h at 37°C. **(D)** EDC34 was incubated with IgG in 1:1 ratio (6 μM) at the indicated time points, or pre-incubated for 30 min with 50 μM of TCEP followed by an additional 24-h incubation step at 37°C. GGL27 in the absence of plasma served a control. **(E)** 6 μM of EDC34 were incubated with IgG Fab or Fc fragments (6 μM) for 30 min at 37°C. Samples were separated on SDS-PAGE and probed with an antibody against CAK27 a C-terminal TFPI-2 peptide region.

Many factors including pH, salt concentrations, or interacting proteins, have been described to alter the antimicrobial activity of a number of AMPs (30). To test whether these parameters have an effect on EDC34, the antimicrobial activity of the peptide was analyzed under five different plasma conditions (including human serum, citrate plasma, lepirudin-, heparin-, and EDTA-plasma, respectively). As shown in Figure 1A, EDC34 is able to completely eradicate the bacteria in citrate plasma (CP), while its antimicrobial activity is decreased in lepirudin or EDTA plasma as well as in serum. When the experiment was performed with heparin plasma the bactericidal effect of EDC34 was significantly blocked resulting in the survival rates rate exceeding 80%. The latter findings were expected since also many other heparin-binding AMPs lose their antimicrobial activity in the presence of heparin (34). Peptide stability can be influenced by many different conditions including proteolytic degradation. To test whether EDC34 is resistant to proteolysis, the peptide was incubated with three different anticoagulant plasmas as well as with serum. Samples were collected after different time points and subjected to western blot analysis probing with an antibody against the C-terminal part of TFPI-2. When incubated with serum, EDC34 was completely degraded after an incubation time of 15 min. However, when the experiment was performed in citrate, lepirudin, or EDTA-plasma, we noticed that EDC34 immediately formed a complex with a protein with an apparent molecular weight of about 180 kDa (Figure 1B). In this complex EDC34 seemed to be more stable as it started to degrade in EDTA plasma after 60 min and remained intact for more than 2 h in citrate and lepirudin plasma.

### EDC34 interacts with immunoglobulins

IgG is one of most abundant proteins in plasma and has a molecular weight in the range of 180 kDa. Thus, we hypothesized that EDC34 can interact with IgG or even with other immunoglobulins. In order to test this hypothesis, EDC34 was incubated with different classes of human purified immunoglobulins, i.e., IgA, IgG, IgE, and IgM and binding was tested by immunoblotting. Our results show that EDC34 avidly binds to all immunoglobulins tested (Figure 1C). When the interaction of the peptide with IgG was studied over time, we found that it was entirely absorbed after a 24 h incubation (Figure 1D). EDC34 contains two cysteine amino acids and to exclude that the interaction between EDC34 and IgG is the result of unspecifically newly formed disulphide bridges, the interaction was further studied under reducing conditions. Moreover, we used a control peptide GGL27 from TFPI-1 (Figure 1D) (35). We next explored whether EDC34 interacts with the Fab or Fc part of IgG. To this end the peptide was incubated with the two fragments. Binding was then assessed by western blot analysis, showing that only the Fc fragment is able to bind to EDC34, further underlining the specificity of this interaction (Figure 1E).

### EDC34-IGG interaction is important to boost complement activation on bacterial surfaces

**Figure 2:**
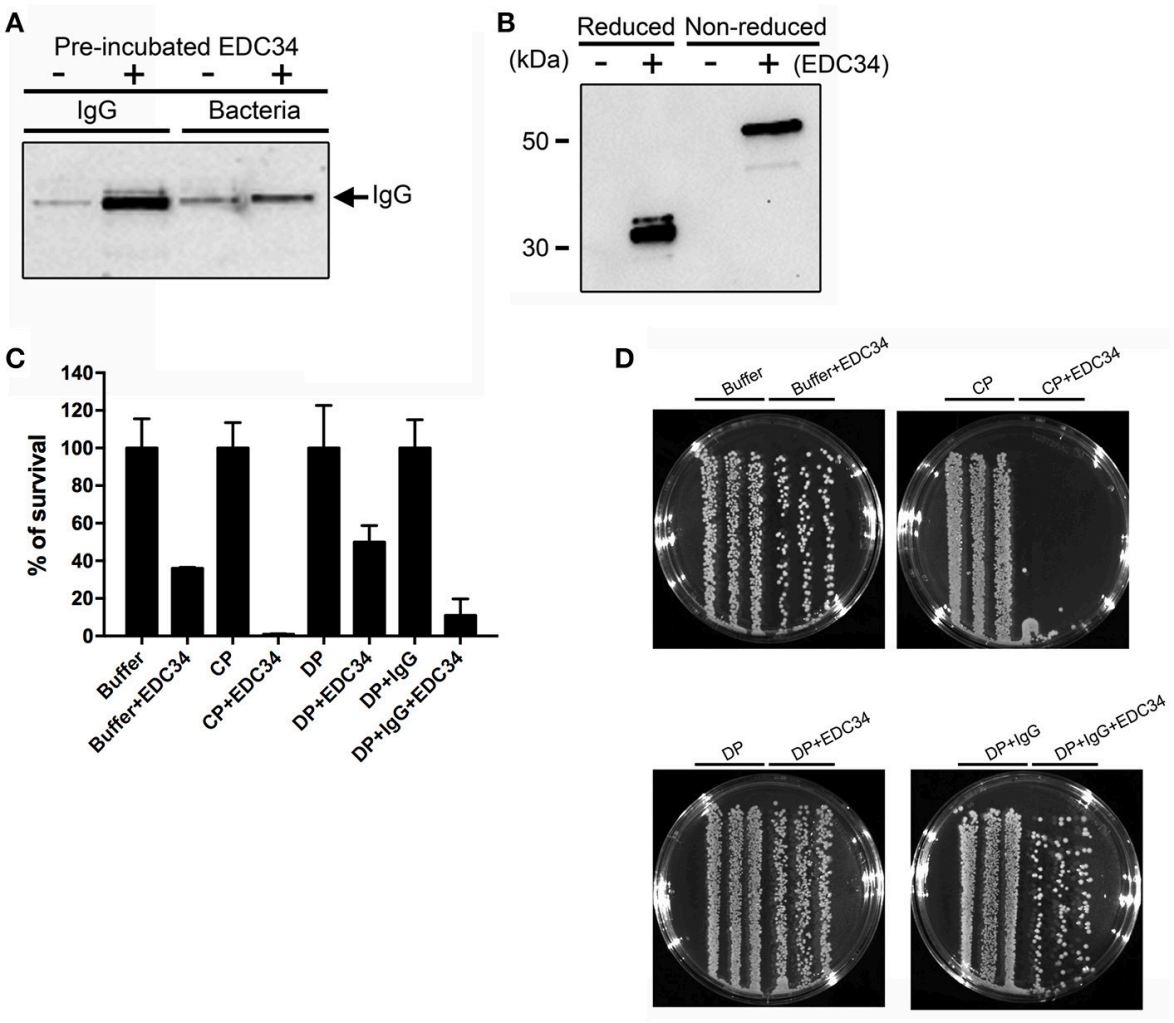
Interaction of EDC34 with IgG enhances C1q deposition on bacterial surfaces. **(A)** EDC34 (2 μM) was incubated with IgG (8 μM) for 24 h in PBS buffer. *E. coli* bacteria were for 1 h at 37°C. Alternatively, EDC34 was incubated with *E. coli* bacteria and then supplemented with IgG (see also Materials and Methods). Samples were separated on SDS-PAGE followed by immunoblotting, and probed with anti-IgG antibodies. **(B)** *E. coli* bacteria were incubated with citrate plasma supplemented with 10 μM of EDC34 for 1 h at 37°C. Proteins at the bacterial surface were eluted, concentrated by a pull down assay, and subjected to SDS-PAGE followed by immunoblotting. Recovered C1q was immuno-detected with anti-C1q antibodies. **(C)** Using viable count assays, antimicrobial activity of EDC34 against *E. coli* was studied. Bacteria and EDC34 were incubated in a PBS buffer, a PBS buffer supplemented with 25 % citrate plasma (CP), a PBS buffer supplemented with 25% immunoglobulin-depleted citrate plasma (DP), or a PBS buffer supplemented with 25% immunoglobulin-depleted citrate plasma reconstituted with IgGs. Mean with SD are shown (*n* = 3). **(D)** Colony cultures of *E. coli* bacteria grown in the indicated conditions shown in petri dishes.

**Figure 3:**
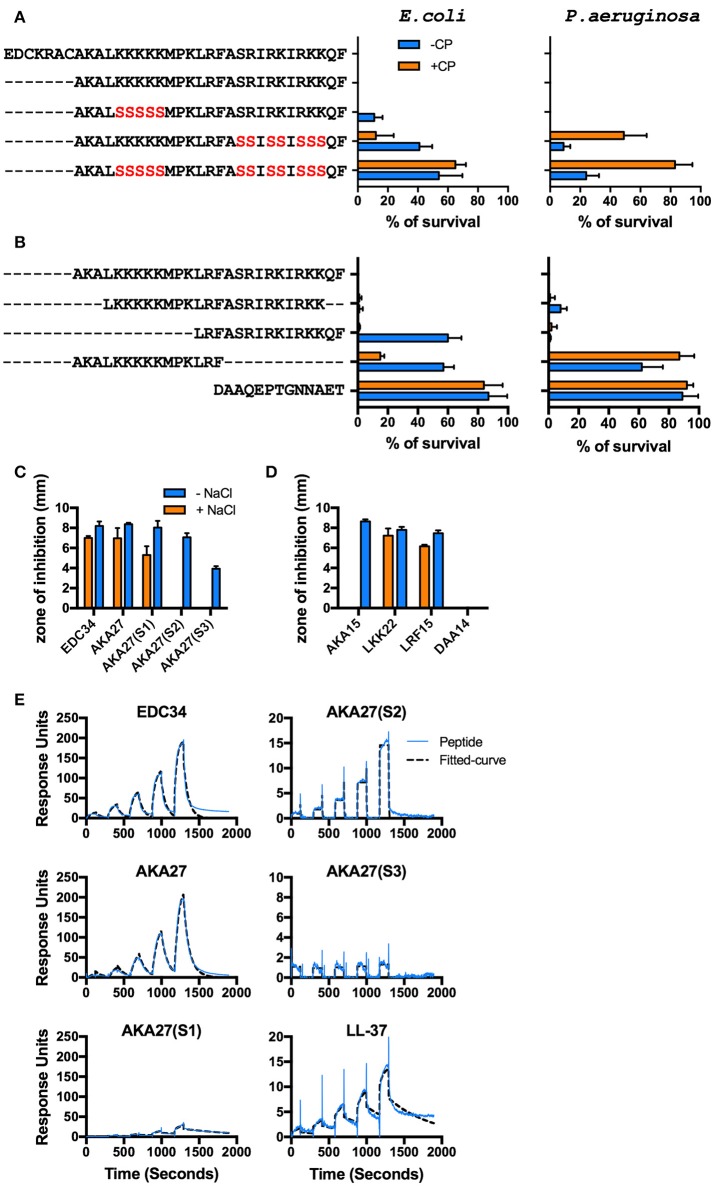
Mapping of EDC34 epitope. **(A,B)** C-terminal TFPI-2 derived peptides at indicated concentrations were incubated with *E. coli* and *P. aeruginosa* bacteria in the presence or absence of 20% citrate plasma, and their antimicrobial effects were measured in viable count assays. **(C,D)** The zone of inhibition of each peptide (X-axis) against *E. coli* bacteria was measured in the presence or absence of 150 mM NaCl using radial diffusion assays (RDAs). DAA14 was used as a negative control. Mean with SD is shown (*n* = 3). **(E)** Surface plasmon resonance analysis of the interaction between IgG and the indicated peptides in the concentration range of 25–400 nM is shown.

To investigate whether EDC34 can alter the antigen-antibody interactions during infection, human IgG was pre-incubated with EDC34 and mixed with *E*. *coli* bacteria. Figure 2A shows that the interaction of EDC34 with human IgG triggered increased opsonization of immunglobulin on the bacterial surface. This effect was less pronounced when EDC34 was first preincubated with *E. coli* bacteria and then later mixed with human IgG (Figure 2A). We next tested whether the binding of EDC34 to IgG can promote activation of the classical pathway of complement. To this end we focused on C1q, as the protein is part of the first subcomponent of the classical complement pathway (36). When EDC34 was added to citrate plasma for 1 h we observed a recruitment of C1q to the surface of *E. coli* bacteria, which was not seen when plasma alone was incubated with the bacteria (Figure 2B). This effect was not dependent on the conditions (reducing or non-reducing) that were chosen. Together the results show that EDC34 is needed for the assembly of C1q at the bacterial surface, which is in line with our previous findings (27). Having shown that EDC34 promotes IgG-mediated opsonization of C1q at the surface of *E. coli* bacteria, we next tested whether this interaction can trigger bactericidal activity. Figure 3C shows that the incubation of EDC34 with bacteria in PBS exerts a moderate killing activity, while almost 100% killing was noted in citrate plasma. Bacterial survival was increased when immunoglobulin-depleted plasma was used and upon reconstitution with IgG the depleted plasma retained its full antimicrobial activity in the presence of peptide (Figures 2C,D). These results demonstrated the important role of EDC34 in boosting complement-mediated bacterial killing.

### Mapping of the antimicrobial and complement-activating site in EDC34

In order to map the epitope responsible for antimicrobial activity, the bactericidal effect of EDC34 and shortened versions of the peptide (Table 1) were tested using *E. coli* and *P. aeruginosa*. To exclude a possible influence of disulphide bonding, a truncated peptide (AKA27) was employed lacking the first seven amino acids, including two cysteine residues. Notably, AKA27 consists of several patches with clustered positively charged amino acids. Earlier studies have demonstrated these amino acids play an important role for the antimicrobial activity of many AMPs (37). To test whether this also applies to AKA27, arginine and lysine residues of the peptide were sequentially replaced by a neutral amino acid (serine) as depicted in Table 1. While AKA27(S1) remained its full antimicrobial potential in 20% human plasma, the bactericidal activity was decreased when AKA27(S2) or AKA27(S3) were analyzed under the same experimental conditions (Figure 3A). To further characterize the active site, truncated versions of AKA27 were synthesized. The sequences of these peptides (LKK22, AKA15, and LRF15) as well as the sequence of a control peptide (DAA14), derived from the N-terminal region of TFPI-2, are listed in Table 1. As shown in Figure 2B, LKK22, spanning the central core region of AKA27, and LRF15, lacking the N-terminal portion of AKA27, retained their antimicrobial activity, when incubated with bacteria in the presence of plasma. However, AKA15, lacking the C-terminal part of AKA27, and the control peptide (DAA14) had no antimicrobial activity under these conditions (Figure 3B). The same activity pattern was recorded using radial diffusion assays (RDAs). Here the antimicrobial activity of the peptides was tested under low and high salt conditions. While in the absence of salt all peptides with the exception DAA14 (negative control) had full or some antimicrobial activity, only EDC34, AKA27, AKA27(S1), LKK22, and LRF15 were able impair bacterial growth under high salt conditions (Figures 3C,D). These findings suggest that the salt concentration might be in part responsible that AKA27(S2), AKA27(S3), and AKA15 have lost their antimicrobial activity in plasma. Finally, we determined the affinity of EDC34, AKA27, AKA27(S1), AKA27(S2), and AKA27(S3) for human IgG. LL-37 was used as control. SPR analysis were performed by running the peptides over an IgG coted chip. As depicted in Figure 3E, EDC34 and AKA27 have highest affinity for IgG, while the affinity of other peptides showed was significantly lower (Figure 3E). Together these findings support the notion that the C-terminal region of AKA27 is important for the interaction with IgG and subsequent activation of the classical complement pathway.

### TFPI-2 is highly expressed during infection and inflammatory conditions

**Figure 4:**
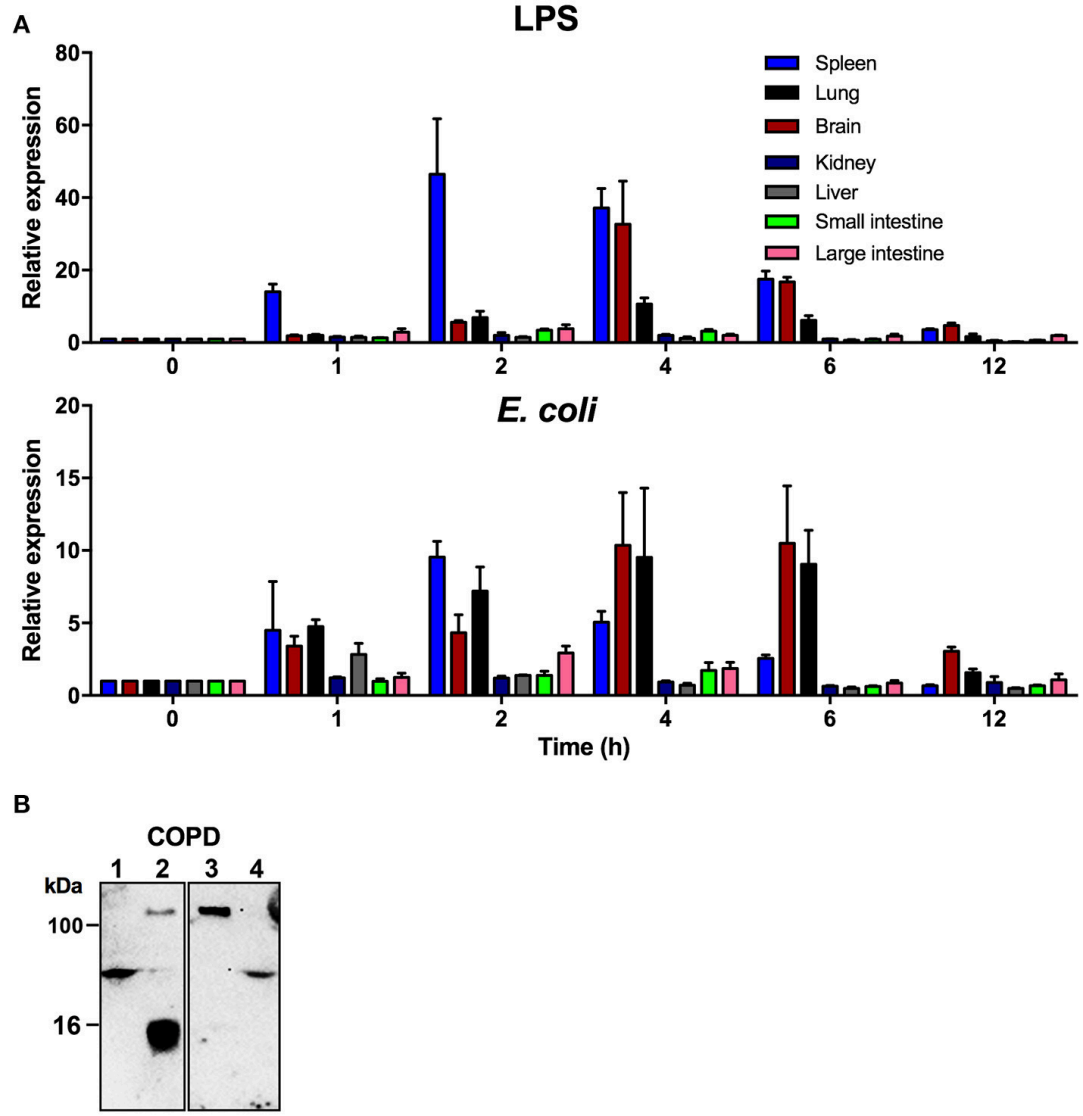
TFPI-2 is highly expressed during endotoxin-induced inflammation and sepsis. **(A)** To measure TFPI-2 expression levels in various organs during inflammation and systemic bacterial infection *TFPI-2*^+/+^ mice were intraperitoneally injected with either 8 mg/kg LPS or *E. coli*. TFPI-2 mRNA expression levels was measured at indicated hours post-injection in spleen, lung, brain, kidney, liver, small intestine and large intestine (*n* = 3). Expression is presented as relative gene expression levels in relation to housekeeping gene *β*-actin. The data shown mean ± SEM. **(B)** Identification of TFPI-2 in COPD patient sputum samples (Lane: 1-4). Cleavage fragments of TFPI-2 were analyzed by immunoblotting using polyclonal antibodies against C-terminal EDC34 peptide.

In order to explore the function of TFPI-2 during systemic inflammation we analyzed the expression levels of TFPI-2 in two relevant animal models, a murine endotoxin-induced model and an *E. coli* infection model. TFPI-2 expression was investigated in spleen, lung, brain, small, and large intestine, liver and kidney in challenged/infected or untreated wild type mice using real-time quantitative PCR (Figure 4A). The results showed that TFPI-2 mRNA expression was induced by 10-fold in the spleen already 1 h post-injection of LPS and peaked at 2 h followed by a transient decrease until 12 h post-injection. The second most effected organ in terms of TFPI-2 expression was the brain showing a peak of expression 4 h post-injection. The same was found for lungs. In addition, we examined kidney, large and small intestine and liver which all showed an additional increase in TFPI-2 expression upon systemic inflammation induced by LPS from *E. coli*. The expression was normalized to time-point 0 h and where expression was 1 time fold change in relation to house- keeping gene *β****-****actin*.

We have previously showed that TFPI-2 is highly expressed and that C-terminal fragments are present in chronic skin wounds. In addition to the previous human and current TFPI-2 expression mouse data, we explored whether C-terminal fragments of TFPI-2 are present in sputum samples from COPD patients. Western blot analysis using polyclonal antibodies against the C-terminal part of TFPI-2 shows that TFPI-2 is highly expressed and that C-terminal fragments are indeed associated with immunoglobulins in sputum from a COPD patient (Figure 4B).

### TFPI-2 is protective during systemic gram-negative infection *in vivo*

**Figure 5:**
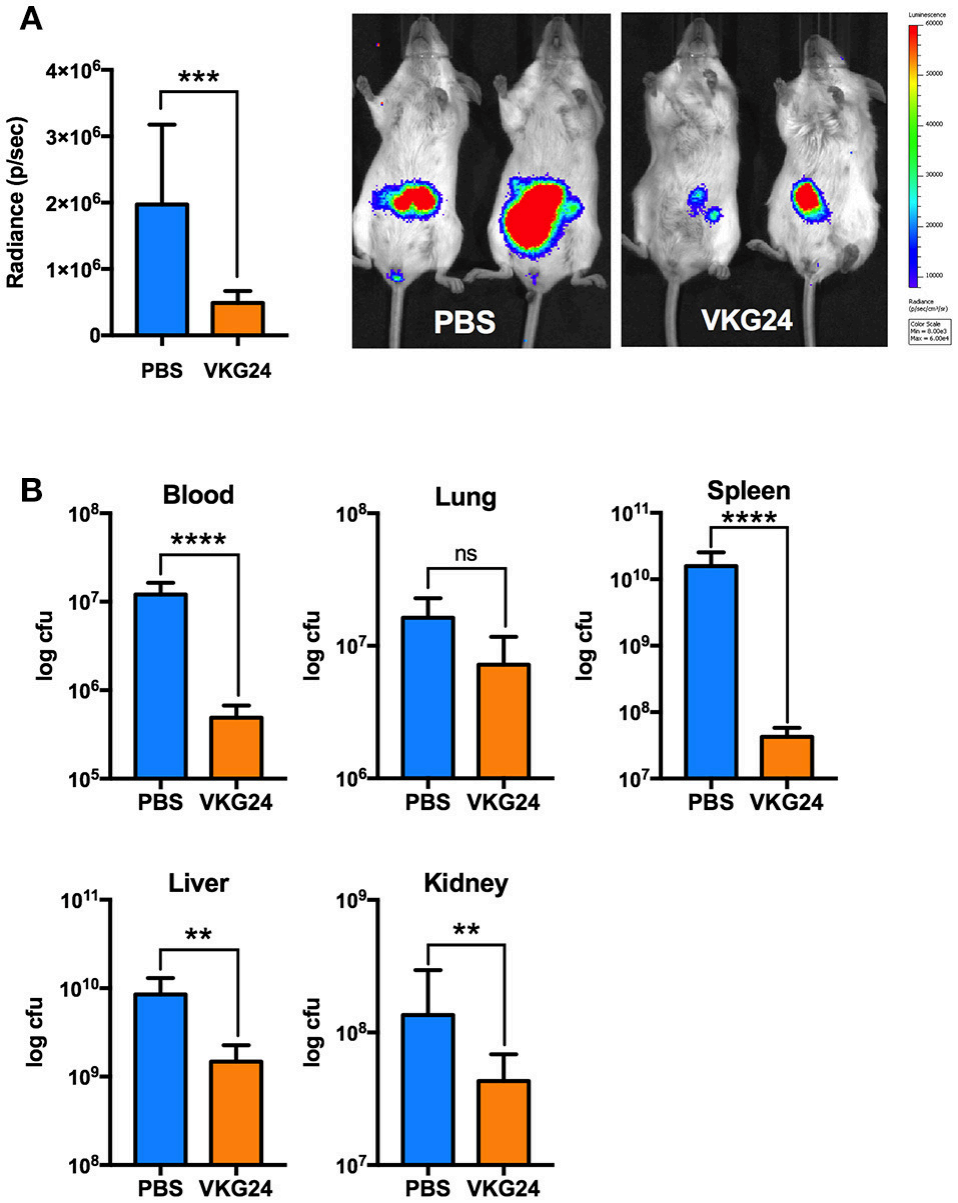
Mouse TFPI-2 peptide VKG24 protects mice from *E. coli*-induced sepsis. Mice were infected with 100 μl (8–8.5 × 10^8^ cfu/ml) *E. coli* bacteria. One hour after infection animals were treated (i. p.) with PBS and VKG24 (500 μg per mouse) for 8 h. Mice (*n* = 5) were then anesthetized and injected (s. c.) with L-012 dye to detect ROS production. IVIS® Spectrum with Life Image® version 4.4 was used for imaging. **(A)** ROS production in radiance (p/sec) for control mice (PBS) and mice treated with VKG24 (PBS & VKG24 *n* = 5). Representative IVIS® Spectrum images indicating ROS production from two control (PBS) and treated (VKG24) mice are included. **(B)** After the IVIS examination, mice were scarified and organs were harvested for cfu count measurements (*n* = 5; liver **p < 0.0048, kidney **p < 0.0036, spleen ****p < 0.0001, ***p < 0.0007, blood ****p < 0.0001).

In the last series of experiments, we employed *in vivo* models to explore both therapeutic and physiological aspects of our findings. To this end, we infected mice intraperitoneally with *E. coli* bacteria. One hour after the bacterial challenge, mice were treated with buffer or the mouse-derived TFPI-2 C-terminal peptide VKG24, a mouse analog of human EDC34. After an 8-h incubation, IVIS® Spectrum images were taken to detect ROS production. Mice were then sacrificed and blood, kidney, lung, liver and spleen were collected to determine bacterial dissemination. As shown in Figure 5A, ROS production in VKG24-treated mice was noticeably decreased when compared with buffer-treated mice. In accordance with these results, we also noticed that bacterial spreading to the different organs was reduced in peptide-treated mice when compared to the controls (Figure 5B).

**Figure 6:**
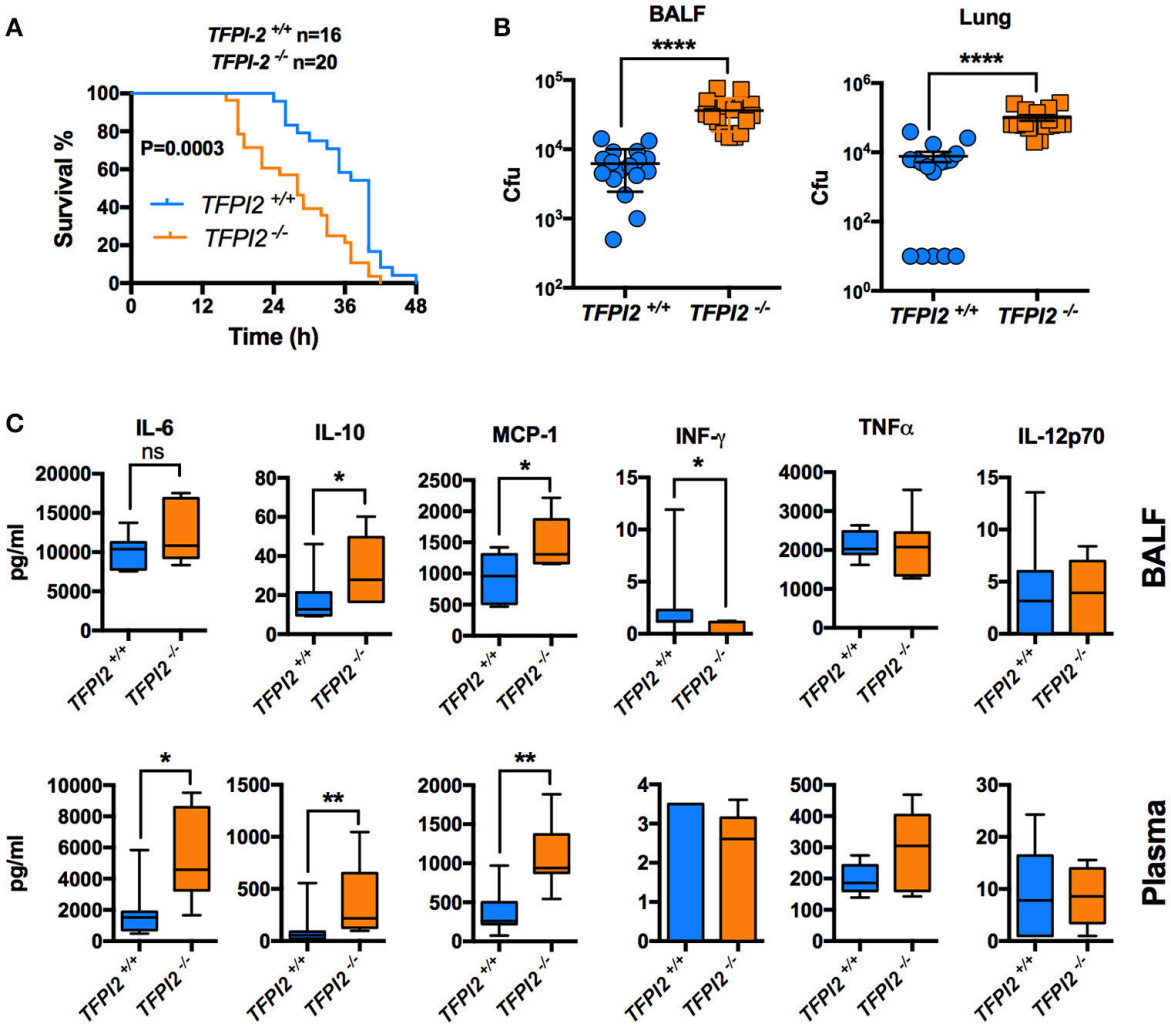
TFPI-2^−/−^ mice are susceptible to acute *Pseudomonas aeruginosa* lung infection. **(A)** Survival. Fifty microliter of *P. aeruginosa* Xen41 bacteria (3–4 × 10^8^ cfu/ml) were administered intra-nasally (i.n.) into *TFPI-2*^+/+^ and *TFPI-2*^−/−^ mice. Survival of the mice was monitored every 2 h. The data is pooled from three different experiments with a total number of 16 wild type mice and 20 *TFPI-2*^−/−^ mice. **(B)** BALF and lung tissue was harvested, placed on ice, homogenized (lung tissue), and colony-forming units (cfu) was determined 12 h after infection (*n* = 17). **(C)** Cytokines were analyzed in BALF and plasma (*n* = 7).

**Figure 7:**
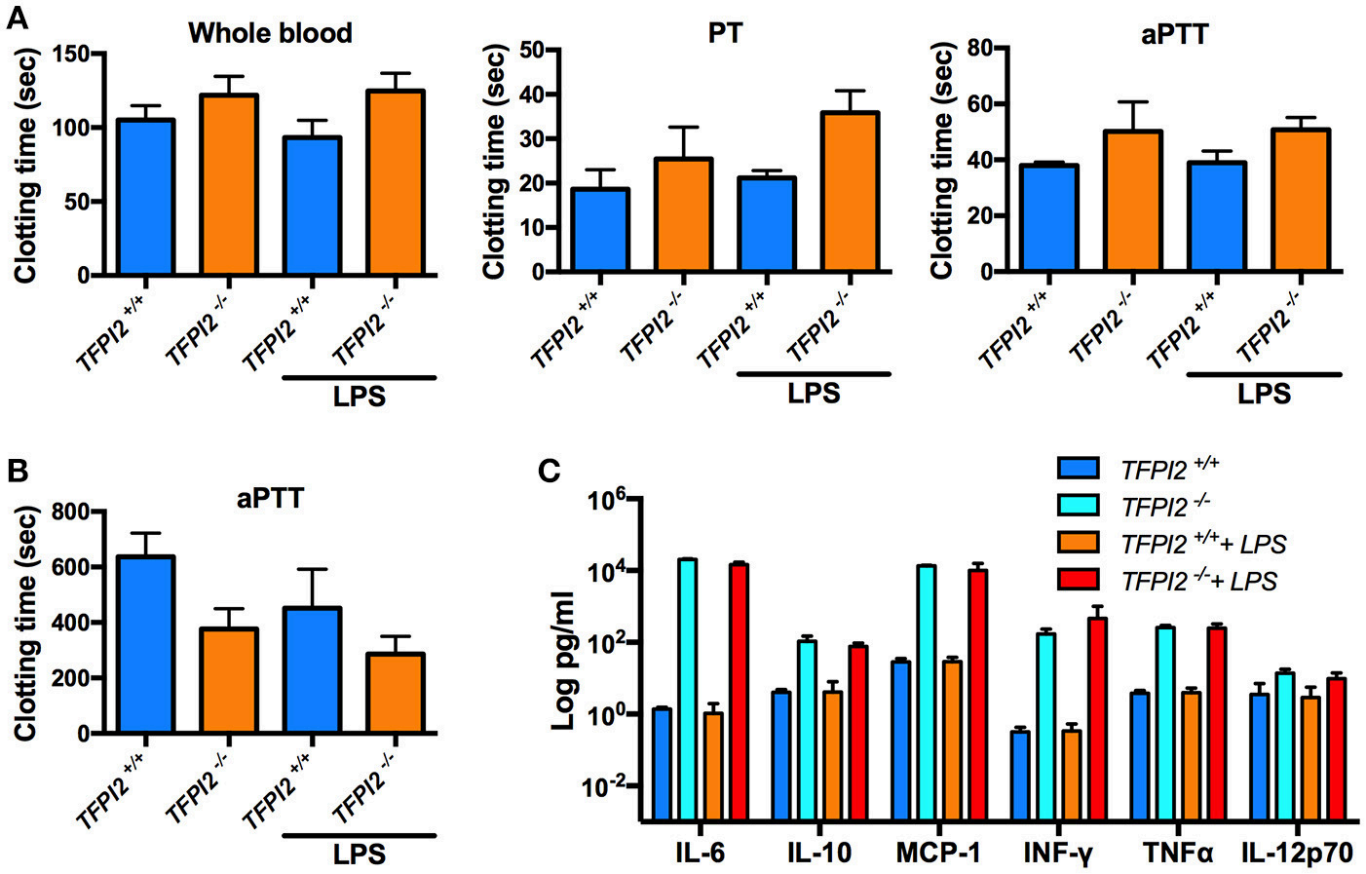
*In vivo* TFPI-2 has no significant impact on coagulation. Mice were intraperitoneally injected with either 8 mg/kg LPS, clotting time, platelets and cytokines were measured after 6 h post-LPS challenge. **(A)** Clotting times was measured in whole blood. Extrinsic pathway, prothrombin time (PT) and the intrinsic pathway activated partial thrombin time (aPTT) was determined in *TFPI-2*^+/+^ and *TFPI-2*^−/−^ citrate plasma. **(B,C)** Total number of platelets counts and indicated cytokine levels were analyzed in blood from *TFPI-2*^+/+^ and *TFPI-2*^−/−^ mice. No significant differences were observed in between the groups.

Given the high expression of TFPI-2 in the lungs during inflammation, we next wanted to pinpoint the role of TFPI-2 in the respiratory tract. We performed intranasal *P. aeruginosa* infection using *TFPI-2*^−/−^ and *TFPI-2*^+/+^ mice. *TFPI-2*^−/−^ mice exhibited a significantly lower survival rate compared to wild type mice (*p* = 0.003) (Figure 6A). In addition, bacterial load was measured in both bronchioalveolar lavage fluid (BALF) and lung tissue was measured after 12 h post-infection (Figure 6B). Indeed *TFPI-2*^−/−^ mice exhibited larger amounts of bacteria in lung tissue compared to wild type mice. Hence, *TFPI-2*^−/−^ mice are more prone to *P. aeruginosa* induced lung infection. To evaluate if the full-length protein of TFPI-2, in addition to limit bacterial infiltration, exerts host defense properties by keeping inflammation at bay during infection we measured inflammatory cytokine levels in both BALF and citrate plasma after 12 h post-infection. On a systemic level (plasma) 12 h post-infection IL-6, IL-10, and MCP-1 was higher in *TFPI-2*^−/−^ mice and no significant changes were observed in INF-γ, TNFα, and IL-12p70 (Figure 6C), whereas in BALF fluid, IL-10, and MCP-1 was higher in *TFPI-2*^−/−^ mice. To rule out the possible anticoagulant activity role of TFPI-2 in TFPI-2^−/−^ mice, we induced LPS mediated septic shock in *TFPI-2*^+/+^ and *TFPI-2*^−/−^ mice and determined clotting times, platelet counts and cytokines in whole blood after 6 h post-LPS challenge. Measurements of clotting times upon endotoxemia did not reveal any difference between *TFPI-2*^+/+^ and *TFPI-2*^−/−^ mice (Figure 7A). No significant differences were observed in platelet counts (Figure 7B), cytokine levels between *TFPI-2*^+/+^ and *TFPI-2*^−/−^ animals (Figure 7C), indicating that TFPI-2 is not involved in anticoagulation and inflammation. Together our results demonstrate a previously undisclosed mechanism based on boosting of complement-dependent immune response to infection. A protective role of TFPI-2 was demonstrated during lung infection by employing TFPI-2^−/−^ and wild type mice. By using an animal model of infection and treatment with a C-terminal mouse homolog of EDC34, we found that our findings can be used for the development of novel antimicrobial therapies.

## Discussion

In this work we present a novel mechanism mediated by TFPI-2 C-terminal peptides to promote complement mediated bacterial killing. In addition, using *TFPI-2*^−/−^ mice we for the first time show a protective role of TFPI-2 during Gram-negative infection. Studies using artificial membranes and live organisms have illustrated that the vast majority of AMPs exert their antimicrobial activity by permeabilizing the membrane of the pathogen. Thus a mechanism, leading to a burst of the bacterial cell wall, has been described for instance for defensins (38, 39), bactenecins (40), and magainins (41, 42). However, there are also other mechanisms, such as the breakage of single-strand DNA by defensins (43), production of hydrogen peroxide by magainins (44), or induction of apoptosis by lactoferricin (45) that can in addition contribute to an elimination of the pathogen. Based on these findings we hypothesized that EDC34 utilizes different strategies to exert its antimicrobial activities. This assumption was based on previous studies showing the peptide has multifunctional properties, as it not only displays antimicrobial activity, but it can also activate the classical complement system and the intrinsic pathway of coagulation (27, 28).

The complement system is activated by three distinctive routes, known as the classical, alternative, and lectin pathway, respectively. Though all three cascades lead to the generation of the same effector functions, only the classical pathway requires the opsonization of immunoglobulins at the bacterial surfaces (46). Recent work has shown that EDC34 can amplify the classical pathway (28). However, the underlying mechanisms were not addressed in previous studies. Here we show that EDC34 exerts its immunomodulatory effect by binding to immunoglobulins which then helps to augment complement activation. Though the interaction of C1q with antibodies is normally needed during this process, it can also occur in an antibody-independent mechanism. To this end C1q binds directly to the surface of the pathogen where it triggers structural changes in the C1s/C1r complex. This then leads to production of C3a and C3b molecules that play a critical role in inflammation, bacterial killing, and phagocytosis (46). To initiate classical pathway C1q has to bind with its CH_2_ domain to the Fc region of IgG (47, 48). Our results show that the interaction of EDC34 with the Fc region of IgG enhances the binding of immunoglobulin to C1q which then in turn leads to an increased activation of the classical complement pathway. Notably, the interaction of EDC34 with the complement system appears crucial since the endogenous antimicrobial activity of the peptide was not sufficient for a complete eradication of the invading pathogen.

TFPI-2 is mainly expressed in brain, spleen and lung during sepsis and LPS-induced systemic inflammation in mice (C57BL/6). Surprisingly, *TFPI-2*^−/−^ mice did not show any significant impact on coagulation during endotoxin-induced sepsis. This is contradictory to the previously claimed functional relevance as an endogenous anti-coagulant, similar to the highly homologous protein TFPI-1 (49). Platelets are well established as a cell type that maintains hemostasis and thrombus growth. Rather than an anti-coagulant, TFPI-2 display characteristics of a host-defense protein with a possible wide variety of functions based on it's expression in the different organs. Based on the reported data we have for the first time shown that the TFPI-2 plays a prominent role in host defense against Gram-negative lung infection in mice. TFPI-2 could therefore constitute an important acute phase reactant in lung tissue with bactericidal and immune-regulatory functions. Based on the detection of proteolytically digested fragments of TFPI-2 found in sputum from patients with COPD, our data further implies that proteases can generate TFPI-2 C-terminal fragments with host defense activities. Such investigations, along side of monitoring of correlations to enzymatic activity and clinical outcome, would likely generate implications for novel treatment routes for COPD patients.

As a proof of concept from a therapeutic perspective, we employed an animal model of infection using VKG24, the murine TFPI-2-derived homolog to EDC34 (27). Previous work has shown that VKG24 exerts, like EDC34, anti-inflammatory activity and protects mice from fatal LPS shock (50). It was further reported that VKG24 exhibits antimicrobial killing activity against *E. coli* under *in vitro* conditions (50). Based on these findings we decided to test the effect of VKG24 in an *E. coli* sepsis model. Our results show that bacterial growth was reduced in blood, liver, kidney, spleen, and lungs when infected mice were treated with VKG24. Along these lines we also noted that ROS production was notably lower in these mice, which indicates that VKG24 exerts an antimicrobial activity by lowering inflammation at the site of infection. Our current study describes increased cytokine levels detected in *TFPI-2*^−/−^ bacterial challenged mice. This is in line with previously published study where we showed reduced cytokine levels in LPS challenged mice treated with VKG24 peptide (50), indicating potent immunomodulatory activity of C-terminal TFPI-2 region against Gram-negative infections. In conclusion, our data show that EDC34 possesses therapeutic potential, as application of the peptide or its murine homolog not only can kill bacteria by permeabilizing their membranes, but also by boosting activation of the classical complement pathway (27). *In vivo* experiments further reveal the peptide motif exerts a systemic bacterial killing activity combined with a non-pathological inflammatory response, which in combination provides a promising approach for future therapeutic applications. Taken together, the evidence presented in this work demonstrates that TFPI-2 is mainly a host-defense protein, while the anti-coagulative functions likely play a minor role in normal hemostasis.

## Author contributions

PP and AS designed the research. MA, GK, ME, SA, SJ, and PP performed the experiments. AE, HH, AS, and PP contributed analytic tools,reagents,materials,analysis tools. PP analyzed the data and wrote the paper.

### Conflict of interest statement

The authors declare that the research was conducted in the absence of any commercial or financial relationships that could be construed as a potential conflict of interest.

The reviewer AT and handling Editor declared their shared affiliation.
