# Supplementary material for: TFPI-2 Protects Against Gram-Negative Bacterial Infection
Source: Front Immunol. 2018 Sep 11;9:2072. doi: 10.3389/fimmu.2018.02072 (PMC6141739; doi:10.3389/fimmu.2018.02072)
Supplement: Supplementary file 1 [file Data_Sheet_1.DOCX]

**Supplementary information**





**Supplementary figure 1. *TFPI-2* disruption in mice.**  **(A)** TFPI-2 replacement construct and partial restriction map; exon are indicated in 1-5 numbers. **(B)** Southern blot analysis after *EcoR1* digestion. **(C)** In genotyping analysis, the targeted allele gave a 564 kb band, whereas the endogenous allele gave a 325 kb band. **(D)** RT-PCR demonstrating that mRNA expression of TFPI-2 was detected in total RNA derived from the liver of TFPI-2*^+/+^* or *TFPI-2^+/–^* or TFPI-2-/- mice, for control *β-actin* was used.

**List of primers**

**RT-PCR**

mTFPI-2_FW: 5’-ACTATAAATGTATTCTCTGAGGAAGCTA-3’

mTFPI-2_RW: 5’-ATCCAGGTAATAAAAGTTATTCTCAT-3’

mBeta actin_FW: 5’-CTGTGCTATGTTGCTCTAGACTT-3’

mBeta actin_RW: 5’-GAATTGAATGTAGTTTCATGGAT-3’

**Genotyping primers**

Gene specific (endogenous, targeted): 5’-CCTACAGGGCAGAAGCTGTTAAAGTC-3’

NEO (targeted): 5’-GGGCCAGCTCATTCCTCCCACTCAT-3’

Gene specific (endogenous): 5’-TTAGGCCTGGTCTGTGTAGCAGGAC-3’
